# Comparison of differences in performance between pigs whose sires were identified using different selection strategies after experimental infection with PRRSV

**DOI:** 10.1093/tas/txae128

**Published:** 2024-08-23

**Authors:** Erin A Little, Jenelle Dunkelberger, Daniel Hanson, John Eggert, Michael G Gonda, Michael D MacNeil, Scott Dee

**Affiliations:** Pipestone Applied Research, Pipestone, MN 56164, USA; Topigs Norsvin USA, Burnsville, MN 55337, USA; Pipestone Applied Research, Pipestone, MN 56164, USA; Topigs Norsvin USA, Burnsville, MN 55337, USA; Department of Animal Science, South Dakota State University, Brookings, SD 57007, USA; Delta G, Miles City, MT 59301, USA; Department of Animal, Wildlife and Grassland Sciences, University of the Free State, Bloemfontein, South Africa; Pipestone Applied Research, Pipestone, MN 56164, USA

**Keywords:** disease, index selection, porcine reproductive and respiratory syndrome, resilience, swine

## Abstract

The objective of this study was to evaluate differences in the performance of offspring of boars selected with an index emphasizing resilience and boars selected based on a traditional index, emphasizing feed efficiency and carcass quality (traditional) index vs. a customized (resilience) index. The resilience index was identical to the traditional index, except that extra emphasis was placed on piglet vitality (increased by 66%), growth rate (decreased by 14%), and feed intake (increased substantially by 5,157%). Sows were mated to either boars selected based on the resilience index or boars selected on the traditional index. Weaned offspring were vaccinated for Porcine Reproductive and Respiratory Syndrome Virus (PRRSV) and experimentally infected with PRRSV RFLP 1-7-4 four weeks later. Offspring were allocated to pens (*n* ~ 27 pigs/pen; *n* = 27 pens/group) by sire-selection group for a total of 1,458 pigs in 54 pens. The weight of each pen was recorded on 0, 42, and 110 d postinfection (DPI) and used to calculate average daily gain (ADG), average daily feed intake (ADFI), and feed conversion ratio (FCR). Mortalities were recorded from 0 to 110 DPI and necropsies were routinely performed to characterize pathogens present within the barn. Pigs classified as full value (i.e., >104 kg and void of defects) were slaughtered and hot carcass weight (HCW), backfat, loin depth, and lean weight were obtained from the slaughter plant. Effects of progeny group on performance, carcass characteristics, and mortality rate were estimated with a mixed linear model. Differences between progeny groups in ADG (*P* > 0.27), HCW (*P* = 0.68), backfat (*P* = 0.13), or loin depth (*P* = 0.39), and mortality rate (*P* = 0.29) were not detected. From 0 to 42 DPI, offspring of boars selected based on the resilience index had higher ADFI (0.06 kg/d, *P* = 0.01) and higher FCR (0.12, *P* = 0.01). In summary, results from this study do not support selection of boars for increased feed intake, piglet viability, and robustness in order to prevent losses caused by PRRSV, but selection response was only measured after one generation of male selection. The impact of multiple generations of selection, or the development of an index including traits derived from data collected under disease-challenged conditions should be explored. The data collected for this study are a valuable resource to explore additional genetic selection strategies for enhanced resilience to a multifactorial PRRS challenge.

## Teaser

Boars were selected for increased resilience and their progeny were intentionally infected with Porcine Reproductive and Respiratory Syndrome Virus (PRRSV) and performance was measured. Immediately postinfection, progeny performance reflected selection criteria but wean-to-finish performance did not support increased performance in the face of PRRSV.

## Introduction

Porcine Reproductive and Respiratory Syndrome (PRRS), which is caused by the PRRS virus (PRRSV), continues to be a costly disease for commercial swine producers throughout the world. In 2013, PRRSV was estimated to cost $US 3.00 per marketed pig, including the costs of the disease itself and preventative measures (Holtkamp et al., 2013). The PRRSV can also weaken a pig’s immune response, allowing opportunistic secondary infections to occur (Gómez-Laguna et al., 2013). Many swine producers vaccinate their pigs for PRRSV and practice strict biosecurity measures to prevent the virus from entering their barns. Although these measures decrease viral transmission and clinical signs, full protection from PRRSV has not been achieved. Vaccination, in particular, has been effective at protecting pigs from PRRSV. However, vaccine efficacy varies among herds and depends on many factors, including the genetic similarity between PRRSV strains used in vaccines and the disease-causing viral strains (Lager et al., 1999; Meng, 2000). Vaccination has not been fully efficacious, in part, because of the high mutation rate of the PRRSV (Brar et al., 2014). Continued research into measures to reduce the cost of PRRSV infections is clearly needed.

One potential control strategy is the use of genetic selection to breed pigs that are resilient to PRRSV infection. Genetic selection has been shown to be an effective method to increase production efficiency for weight gain, feed efficiency, litter size, and carcass characteristics. The goal of breeding for enhanced disease resilience is to breed animals capable of maintaining performance or quickly recovering after pathogen exposure (Mulder and Rashidi, 2017). In the context of PRRSV, a resilient pig continues to grow after being exposed to PRRSV and yields a quality, full value carcass.

The objective of this study was to evaluate differences in performance, carcass characteristics, and mortality rate between 2 groups of pigs; pigs sired by boars selected based on either a traditional index, emphasizing feed efficiency and carcass quality (i.e., traditional) index vs. a customized (resilience) index. The resilience index was identical to the traditional index, except that extra emphasis was placed on piglet vitality, as well as feed intake and growth rate during finishing.

## Materials and Methods

Prior to the start of this study, the South Dakota State University and Pipestone Applied Research (PAR) institutional animal care and use committees (SDSU IACUC 18-030A and PAR IACUC 1-18) reviewed and approved the trial protocol, mortality standards, and caretaker handling certification. These guidelines were upheld throughout the trial.

All pigs were sourced from a 5,000-head sow breeding farm located in northeastern South Dakota, USA. All sows were naïve to PRRSV. Each sow was mated using semen from only one sire. Boars were selected based on Topigs Norsvin’s traditional selection index (traditional index; *n* = 11 sires), which emphasizes feed efficiency and carcass merit, or a customized index, developed for this study. This customized index was identical to the traditional index, except that the emphasis placed on piglet vitality was increased by 66%, the emphasis on growth rate was decreased by 14% and the emphasis on feed intake was increased considerably by 5,157% (resilience index; *n* = 11 sires). If a female showed signs of heat the day after being inseminated, she was rebred to the same boar. Females were farrowed in crates. After farrowing the piglets were individually identified using the Caisley tagging system (Caisley Eartag Limited, North Yorkshire, UK). Parentage, foster dam (if any), parity of the dam, birth weight and sex were recorded for each piglet. Cross-fostering was permissible and practiced according to the following specifications: piglets of extreme (small or large) size relative to littermates were candidates for cross-fostering within the first 24 h after farrowing (to limit competition among littermates); however, a piglet could only be moved to another litter of the same genetic group. Litter size was adjusted to match teat count of the sow and thus limit competition amongst littermates. Piglets were weaned between 14 and 29 d of age and received 0.5 mL of Baytril (Bayer Healthcare LLC). Average age at weaning was 20.3 ± 0.3 and 20.4 ± 0.3 for pigs in the resilience and traditional groups, respectively. At weaning, piglets that appeared healthy, had good leg quality, were free of defects, and weighed more than 3.6 kg were selected for the trial. Selected piglets were moved to a 2,400-commercial research barn located in southwestern Minnesota, USA. At weaning, piglets were moved to a 2,400 head wean-to-finish barn located in southwestern Minnesota, USA. This research barn was tunnel ventilated with 2 rooms consisting of 39 and 42 pens, although only 54 pens (26 and 28 per room) were used for this study. Each room had a separate, but identical, ventilation system (Expert Series, Automated Production) that regulated the environment within the room. Individual and pen wean weights were recorded upon entry into the trial and a radio frequency identification tag (Merck and Co, Rahway, New Jersey, USA) was placed in the ear of each pig. To facilitate processing, pigs were received at the finishing barn over 5 weaning events. Thus, wean weight, wean weigh date, and wean age varied within progeny groups but did not significantly differ between the groups. On the final wean day, all pigs were vaccinated with 2 mL of a PRRS modified-live virus vaccine (Ingelvac PRRSV ATP, Boehringer Ingelheim).

Because pigs were allocated to pens by progeny group, pen was the experimental unit. The trial was designed as a randomized block. Each block (*n* = 27) consisted of 2 adjacent pens, one containing pigs sired by boars of the resilience group and the other containing pigs sired by boars of the traditional group. Block was used to account for environmental variation such as humidity, temperature, air quality, and other environmental factors that may vary by location with a tunnel-ventilated barn. Each pen consisted of 27 pigs, which were randomly selected from the wean group, except that the smallest 27 pigs of each group were placed into a single block in an attempt to limit competition within the pens. Male and female pigs were penned together with an equal sex-ratio in each pen of a block. Each pen, which had 0.65 m^2^ of floor space per pig, was equipped with a 4-hole dry feeder (Crystal Spring Hog Equipment, Ste. Agathe, Manitoba, Canada) with a capacity of 136 kg and 34.93 cm wide feeder hole and 2 cup water dishes. Feed and water were provided ad libitum. An automated feeding system was used for all pens, which recorded the time and amount of feed delivered (Feedlogic Corporation, Willmar, MN).

Four weeks after vaccination, pigs were individually inoculated with the PRRSV lineage 1 strain 174 via intramuscular injection (dose = 2 × 10^3.5^ tissue culture infectious (TCID50) dose) under veterinary supervision. Following the guidelines of a previous PRRS challenge study (Dee et al., 2018), pigs were observed weekly by the attending veterinarian and welfare director at Pipestone Veterinary Clinic to advise on the proper timing and method of antibiotic intervention. Visual assessment of the pigs and their environment, including access to food and water, was completed daily by a caretaker under the direction of the site veterinarian. This included evaluating pig health using the individual pig care (IPC) scoring system (Pineiro et al., 2014) where “A” = mild signs of disease; “B” = medium; “C” = serious; and “D” = very serious or near death. On any given day, pigs that were assigned a score of “B” or “C” were treated with antibiotics and added to a watch list for frequent monitoring. Pigs deemed to be immobile and unable to eat or drink were euthanized by a qualified caretaker who had been trained by the Pipestone Welfare Department and veterinarian. Once 20% of the population was classified as either a “B” or “C”, mass medication was administered. This occurred twice; first at 21 d postinoculation (DPI) when each pig received a 1 mL intramuscular (IM) injection of Excede (ceftiofur crystalline free acid, Zoetis, Parsippany, NJ, USA) to address respiratory distress, and Predef (Zoetis, Parsippany, NJ, USA) was administered to address inflammation. Pigs were mass-medicated a second time at 26 DPI via water-soluble LinxMed (Lincomycin Hydrochloride Powder, Bimeda, Oakbrook Terrace, IL, USA) at a dose of 160 g per 2 gallons of freshwater stock solution for 7 d, to address signs of pneumonia and arthritis.

Oral fluid samples were collected weekly starting at −7 DPI using a cotton rope located in each pen. These samples were sent to the Animal Disease Research and Diagnostic Laboratory (ADRDL, Brookings, SD, USA) at South Dakota State University and tested for PRRSV and influenza. If the sample tested positive for PRRSV prior to challenge, the virus was sequenced to confirm that the identified strain matched the vaccine strain. Pigs that died were necropsied by a trained caretaker and samples were submitted to the South Dakota State University ADRDL to monitor the presence of pathogens throughout the study.

Body weight and feed intake were recorded on a pen basis and summarized at days 0, 13, 42, and 110 DPI to calculate average daily gain (ADG), and average daily feed intake (ADFI) for the periods. Feed conversion ratio (FCR) was calculated from these data. Pigs were marketed at a target weight of 127 kg. The first marketing event occurred at 111 DPI and continued over a 5-wk period. Both genetic groups averaged 156 d on feed. Pigs sent to the packing plant weighing more than 104 kg and void of defects (e.g., umbilical hernias and intact males) were classified as full value (FV). Pigs weighing less than 104 kg or with a defect were sent to a secondary market and classified as a Light Cull and Defect Cull, respectively. The FV pigs were harvested at a packing plant where hot carcass weight (HCW), percent lean, and depths of subcutaneous fat and longissimus muscle were recorded. Individual weights were collected on the morning of marketing and HCW was recorded during harvesting.

All analyses were performed using R software (R Core Team, 2021). Pen was the experimental unit. The pen means for incidences of FV, light, defect, and mortality; postweaning ADG, DFI, FCR, and the carcass data were the dependent variables. The model used to analyze the data was:


$$\begin{array}{l} y_{ijk}=\mu +G_{i}+B_{j}+e_{ijk} \end{array}$$

In this equation, $y_{ijk}$ represents the mean of the dependent variable for pigs in the *k*th pen, µ represents the overall mean, $G_{i}$ represents the *i*th progeny group (i.e., pigs sired by boars selected based on the resilience or traditional index), and $B_{j}$ represents the *j*th random barn location (block with 27 levels) in the barn. Weight on day 0 (i.e., initial weight) was included in the model for ADG between days 0 and 42 DPI and for FCR. A second linear mixed model was used to estimate effect of progeny group on birth weight and ADG from birth to weaning, where the individual piglet was the experimental unit. The data were analyzed using the model:


$$\begin{array}{l} y_{ijkl}=\mu +\;G_{i}+S_{j}+\;M_{ik}+e_{ijkl} \end{array}$$

In this equation, $y_{ijkl}$ represents either birth weight or ADG from birth to weaning of the *l*th individual piglet, µ represents the overall mean, $G_{i}$ represents the fixed effect of the *i*th progeny group, $S_{j}$ represents the fixed effect of the *j*th sex of the piglet (barrow or gilt), and $M_{ik}$ represents the random effect of *k*th dam nested within the *i*th group of each piglet. Interaction effects of birth date*sex and progeny group*sex were not statistically different for either trait (*P* > 0.10) and thus were not included in the final model. The value *P* ≤ 0.05 was used as the criterion for declaring effects significant.

## Results

Pigs in the traditional group tended to be heavier at birth than pigs in the resilience group (*P* = 0.06; Table 1). Males were 0.05 kg heavier at birth than females (*P* < 0.01). No difference in weaning weight, weaning age, or ADG (birth-to-weaning) was detected between progeny groups (*P* = 0.65, *P* = 0.86, and *P* = 0.95, respectively). Only the vaccine strain of PRRSV was found in pigs prior to 0 DPI, suggesting that pigs were not exposed to wild-type PRRSV before being challenged with PRRSV lineage 1 strain 174.

**Table 1.** Least squares means for the effect of progeny group (pigs sired by boars selected based on the resilience vs. traditional index) for traits evaluated from birth to weaning

	Progeny group		
Variable	Resilience	Traditional	*P* value
Pigs, *n*	727	730	
Birth weight, kg	1.45 ± 0.36	1.52 ± 0.30	0.06
Average daily gain, kg/d	0.22 ± 0.01	0.22 ± 0.01	0.95
Pens, *n*	27	27	
Weaning weight, kg	6.15 ± 0.13	6.20 ± 0.13	0.65
Weaning age, d	20.33 ± 0.3	20.38 ± 0.3	0.86

From weaning to harvest, no differences in growth rate and ADFI were detected between the groups (*P* = 0.27, 0.11; Table 2). However, the traditional group was more efficient than those in the resilience group (*P* < 0.01), likely as a consequence of the numerical advantages in both ADG and ADFI of pigs in the traditional group.

**Table 2.** Least squares means for the effect of progeny group (pigs sired by boars selected based on the resilience vs. traditional index) for traits evaluated postweaning

	Progeny group		
Variable	Resilience	Traditional	*P* value
Pens, *n*	27	27	
Weaning to Harvest			
Average daily gain, kg/d	0.76 ± 0.01	0.77 ± 0.01	0.27
Average daily feed intake, kg/d	1.87 ± 0.02	1.84 ± 0.02	0.11
Feed conversion ratio	2.45 ± 0.01	2.39 ± 0.01	<0.01
PRRSV-challenge period (0 to 42 d postinoculation)			
Average daily gain, kg/d	0.56 ± 0.01	0.56 ± 0.01	0.82
Average daily feed intake, kg/d	1.22 ± 0.03	1.16 ± 0.03	0.01
Feed conversion ratio	2.20 ± 0.02	2.08 ± 0.02	0.01

During the 42 d challenge period, no difference in ADG of pigs was detected between the groups (*P* = 0.82, Table 2). This similarity in growth rate was achieved despite the resilience pigs having consumed 0.6 kg more feed per day than the traditional pigs (*P* < 0.01). Thus, traditional pigs had a lower (*P* < 0.01) FCR than the resilience pigs.

Differences in live weight at harvest, HCW, percent yield, subcutaneous fat depth, and longissimus muscle depth were not detected between the traditional and resilience groups (Table 3, *P* > 0.05). Pigs that were sired by boars from the T line had a significantly greater predicted % lean (*P* = 0.02). Further, pigs that died, presented defects, or failed to attain the desired minimum weight for harvest represent opportunity costs, but no differences were detected between the groups for the final outcome classifications (Table 4).

**Table 3.** Least squares means for the effect of progeny group (pigs sired by boars selected based on the resilience vs. traditional index) for carcass traits

	Progeny group		
Variable	Resilience	Traditional	*P* value
Live weight at harvest, kg	124.37 ± 0.43	124.72 ± 0.43	0.33
Hot carcass weight, kg	92.60 ± 0.36	92.80 ± 0.36	0.68
Yield, %	74.4 ± 0.01	74.2 ± 0.01	0.17
Subcutaneous fat depth, mm	17.5 ± 1.9	17.1 ± 1.9	0.13
Longissimus muscle depth, mm	63.2 ± 4.8	63.7 ± 4.8	0.39
Lean, %	55.1 ± 0.2	55.7 ± 0.2	0.02

**Table 4.** Estimates for the effect of progeny group (pigs sired by boars selected based on the resilience vs. traditional index) for traits evaluated at marketing

	Progeny group		
Final Outcome^1^	Resilience	Traditional	*P* value
Full value, %	81.8 ± 0.02	83.7 ± 0.02	0.39
Mortality, %	16.2 ± 0.02	14.4 ± 0.02	0.29
Defect culls, %	0.7 ± 0.01	0.5 ± 0.01	0.73
Under-weight, %	1.2 ± 0.01	1.4 ± 0.01	0.79

^1^Final Outcome is a binary outcome assigned to every pig at the end of the study: Full Value: a pig was free of defects and weighed more than 104 kg at slaughter (1), or not (0); mortality: a pig died (1), or not (0), during the wean-to-harvest period; defect cull: a pig was sold to a secondary market due to a defect (1), or not (0); Light Cull: a pig was sold to a secondary market due to low body weight (<104 kg) (1), or not (0).

The source farm was positive for influenza. Therefore, the piglets were positive for influenza when they arrived at the research barn. Prior to challenge, 75% of necropsied pigs were also positive for *Streptococcus suis.* Fecal samples collected from a subset of pens were positive for rotavirus, but that disease was limited to only a small number of pens and no clinical signs of disease were observed prior to 0 DPI. Oral fluids and tissue samples were routinely collected from pigs postinoculation. Results of oral fluid sampling revealed that pigs were positive for PRRSV 1-7-4 at 3, X, and X DPI and for influenza? PCV2? at X and X DPI, respectively. Tissue samples were collected from a select number of pigs that died during the challenge phase. Pathological evaluation of tissue samples revealed the presence of *Actinobacillus suis, Haemophilus parasuis*, and *Pasturella multocida* at X weeks postinoculation and *Bronchopneumonia* and *Escherichia coli haemolytic* at 8 wk postinoculation.

## Discussion

Genetic selection is a tool used in many livestock species to increase the rate of genetic improvement. Feed is the greatest cost for swine producers, thus selection for improved feed efficiency and growth rate are consistent with increased profitability (Niemi et al., 2015) and reduced environmental impact (Hume et al., 2011). In recent years society has become increasingly concerned about incorporating health and welfare issues in more holistic approaches for pork production (Merks et al., 2012; Denver et al., 2023). Taken together these factors encourage inclusion of resilience as a consideration in swine improvement programs (Hermesch et al., 2015).

Globally, PRRS is consistently regarded as one of the most economically devastating diseases impacting swine producers. Since recognition of the virus in the late 1980s, producers have suffered major financial losses due to PRRS (Holtkamp et al., 2013), mainly as a result of decreased performance and increased mortality following infection (Neumann et al., 2005). The severity and loss from a PRRS outbreak varies depending on factors such as PRRSV isolate or the presence of co-infections. Therefore, the objective of this study was to evaluate the possibility of using selective breeding to create differences in performance and mortality among commercial finishing pigs following experimental challenge with a highly pathogenic strain of PRRSV. To do this, 2 different progeny groups were created: pigs sired by boars selected based on a traditional, performance-oriented selection index (the traditional index) or an experimental, customized selection index (the resilience index). The resilience index was identical to the traditional index, except that extra emphasis was placed on piglet vitality (higher by 66%), as well as feed intake (higher by 5,157%) and growth rate (lower by 14%) during finishing.

Piglet vitality was defined as the genetic effect of piglet survival from birth to weaning. Increased weighting was applied to this trait under the assumption that survival to weaning is genetically correlated with WTF survival following disease challenge. However, results from this study show that mortality rate was numerically (2% ± 0.02) higher for pigs in the resilience vs. traditional group. The percentage of FV pigs at the time of harvest is another important metric of robustness. Non-FV pigs either died, were under-weight, or exhibited severe physical defects at the time of marketing. Conversely, FV pigs survived the disease challenge and achieved the minimum harvest weight by the time of marketing. In this study, no significant difference in the percentage of FV pigs was detected between the 2 progeny groups. Each genetic group was comprised of 27 replicates; to achieve 80% power at a significance level of 5%, a 30% difference in the percentage of FV pigs would need to have been observed. Additionally, a 66% increased emphasis on this trait may not be great enough to translate to decreased mortality in the resilience group.

The increased weight placed on feed intake during finishing in the index used to select the sires of the pigs was also predicted to improve resilience to disease challenge because results of other studies suggest that anorexia may occur as a result of decreased feed intake following disease challenge (Li and Patience, 2017). The significant emphasis placed on this trait was observed during the challenge period when pigs in the resilience group had significantly greater feed intake, but ADG was similar for both groups. This is consistent with results for FCR, which showed that pigs in the traditional group had significantly lower wean-to-harvest FCR and greater percent lean than pigs in the resilience group. These results also agree with the way that boars were selected for this study since the increased feed intake had greater emphasis in the selection of the resilience boars, but without regard for better FCR.

Taken together, differences between progeny groups in this study suggest that the combination of increased weighting on piglet vitality, feed intake, and growth rate, did not translate to faster growth or lower mortality following disease challenge. As described above, this could be because our assumptions about the genetic relationship between piglet vitality, feed intake, and growth rate measured under non-challenged conditions with robustness under disease challenge, were incorrect. In other words, different sets of genes may control growth under non-challenged vs. disease-challenged conditions. Results of additional genetic analyses of the data collected from this trial provide evidence of substantial, natural genetic variation in host response to challenge (Dunkelberger et al., 2022). These results indicate that selection for improved mortality and growth rate following disease challenge is possible when using data collected under challenged conditions.

In general, more research is needed to better understand the relationship between selection for enhanced production under non-challenged conditions with robustness under challenged conditions. For example, the resource allocation theory suggests a potential trade-off between selection for production efficiency (i.e., feed efficiency) and the ability to mount an effective immune response (Rauw, 2012). However, results of an experimental challenge study show that pigs divergently selected for increased feed efficiency responded better to PRRSV-challenge than pigs selected for low feed efficiency (Dunkelberger et al., 2015). This may partially explain why pigs in the traditional group exhibited a similar level of robustness to the resilience group following infection. However, more research is needed to understand the relationship between these performance traits with robustness to disease challenge.

Another potential explanation for the lack of significant differences between progeny groups could be that, although sires were selected using 2 different indices, all boars originated from the same population. Since the dams of all the pigs that were evaluated were of the same genetic line and from a single multiplier herd only one-half generation of selection separated the 2 groups. This is likely to be the main reason that the observed differences in performance between the groups were small. Results from other studies show minimal differences between lines following one-half generation of selection (Falconer, 1954; Nielsen et al., 1997; Márquez et al., 2010; Gilbert et al., 2017) in support of this interpretation. The magnitude of the differences that were observed in this study reflect the magnitude of the effects that may be observed by a commercial swine producer using boars from these lines on an unrelated population of females. However, significant differences in performance and robustness have been detected between divergent selection lines after 5 or more generations of selection (Doeschl-Wilson et al., 2009; Faure et al., 2013; Mpetile, 2014; Dunkelberger et al., 2015).

This study was conducted in a commercial research facility and, therefore, representative of field conditions and translatable to commercial production. The use of a polymicrobial disease challenge model, which is reflective of commercial conditions, was a strength of the experimental design. Overall, the average percentage of FV pigs was 83%, indicative of moderate exposure to health challenge (Cornelison et al., 2018), which is a realistic assumption for pigs finished in leading hog-producing states.

Diagnostic testing was routinely performed throughout the study to obtain an overview of pathogens present pre- and postinoculation with PRRSV and results were consistent with the typical pathogen profile following a PRRS break. Yu et al. (2012) reported that highly pathogenic PRRSV accelerates the rate of infection and bacterial load of *Haemophilus parasuis*. Results from this study support this finding. Other pathogens detected in this study include influenza, *Actinobacillus suis, Pasturella multocida, Bronchopneumonia* and *Escherichia coli haemolytic.* To our knowledge, this was the first study to evaluate differences in WTF performance and mortality of pigs following experimental, individual inoculation with PRRSV in a commercial setting.

The use of individual inoculation was also a strength of the experimental design for evaluating differences between genetic groups. Rowland et al. (2012) identified lack of control as a major limitation of field trials for performing disease research. For instance, one of the major challenges of using field data for this purpose is variation in the level of pathogen exposure. While it was not possible to control the level of exposure to all pathogens present in this study, exposure to PRRSV was standardized by inoculating each pig with the same infectious dose of PRRSV, via the same route of exposure, and on the same day. Other attempts to control potential environmental variation in this study included sourcing all pigs from the same sow farm born of the same maternal line. Matings were also balanced by parity by group. Further, progeny groups had the same average wean age and were equally divided among both sides of the research barn at placement. Sourcing pigs to ensure the same average age at placement and, therefore, upon exposure to PRRSV, was critical, given that results of previous studies suggest that even slight differences in age for young pigs can impact PRRS viral load (Cho et al. 2005; 2006). “Block” was also fitted in the model for analyses of most traits in an attempt to account for other environmental differences between groups of pens. Not surprisingly, “block” accounted for substantial variation for analysis of each trait, likely as a result of differences in temperature, air quality, humidity, and other factors known to vary depending on location within a tunnel-ventilated barn. To summarize, extensive effort was invested to control known sources of variation for a disease challenge study, while still assessing pigs in an environment representative of commercial conditions.

## Conclusion

Minimal differences in performance, carcass characteristics, or mortality were detected between pigs sired by boars selected based on the traditional vs. the experimental resilience index that was designed for this study. A potential explanation for this finding could be as simple as the fact that pigs in the 2 groups were the result of only one-half generation of selection. One way to assess this would be to reevaluate differences in performance and mortality following several additional generations of selection. Another potential explanation could be that the combination of traits emphasized in the resilience index are not the most appropriate traits to emphasize to select pigs for enhanced robustness to challenge. It could be argued that, absent appropriate indicator traits, selection for enhanced robustness to disease must be conducted using data collected under diseased challenged conditions.

## References

[CIT0002] Brar, M. S., M. Shi, R. K. Hui, and F. C. Leung. 2014. Genomic evolution of porcine reproductive and respiratory syndrome virus (PRRSV) isolates revealed by deep sequencing. PLoS One. 9:e88807. doi:10.1371/journal.pone.0088807 24698958 PMC3974674

[CIT0003] Cho, J., S. Dee, J. Deen, and K. Faaberg. 2005. The influence of animal age, bacterial co-infection and porcine reproductive and respiratory syndrome virus (PRRSV) isolate pathogenicity on virus load in individual pigs. Proc. Allen D. Leman Swine Conference. Retrieved from the University of Minnesota Digital Conservancy. [accessed August 8, 2022]. https://hdl.handle.net/11299/142573

[CIT0004] Cho, J. G., S. A. Dee, J. Deen, A. Guedes, C. Trincado, E. Fano, Y. Jiang, K. Faaberg, J. E. Collins, M. P. Murtaugh, et al. 2006. Evaluation of the effects of animal age, concurrent bacterial infection, and pathogenicity of porcine reproductive and respiratory syndrome virus on virus concentration in pigs. Am. J. Vet. Res. 67:489–493. doi:10.2460/ajvr.67.3.489 16506914

[CIT0005] Cornelison, A. S., L. A. Karriker, N. H. Williams, B. J. Haberl, K. J. Stalder, L. L. Schulz, and J. F. Patience. 2018. Impact of health challenges on pig growth performance, carcass characteristics, and net returns under commercial conditions. Transl. Anim. Sci. 2:50–61. doi:10.1093/tas/txx005 32289106 PMC7107292

[CIT0007] Dee, S., J. E. Guzman, D. Hanson, N. Garbes, R. Morrison, D. Amodie, and L. G. Pantoja. 2018. A randomized controlled trial to evaluate performance of pigs raised in antibiotic-free or conventional production systems following challenge with porcine reproductive and respiratory syndrome virus. PLoS One. 13:e0208430. doi:10.1371/journal.pone.020843030521587 10.1371/journal.pone.0208430 PMC6283559

[CIT0008] Denver, S., T. Christensen, T. B. Lund, J. V. Olsen, and P. Sandøe. 2023. Willingness-to-pay for reduced carbon footprint and other sustainability concerns relating to pork production – a comparison of consumers in China, Denmark, Germany and the UK. Livest. Sci. 276:105337. doi:10.1016/j.livsci.2023.105337

[CIT0009] Doeschl-Wilson, A. B., I. Kyriazakis, A. Vincent, M. F. Rothschild, E. Thacker, and L. Galina-Pantoja. 2009. Clinical and pathological responses of pigs from two genetically diverse commercial lines to porcine reproductive and respiratory syndrome virus infection. J. Anim. Sci. 87:1638–1647. doi:10.2527/jas.2008-1447 19181772

[CIT0010] Dunkelberger, J. R., N. J. Boddicker, N. V. Serão, J. M. Young, R. R. Rowland, and J. C. Dekkers. 2015. Response of pigs divergently selected for residual feed intake to experimental infection with the PRRS virus. Livest. Sci. 177:132–141. doi:10.1016/j.livsci.2015.04.014

[CIT0011] Dunkelberger, J. R., C. A. Sevillano, E. Little, S. Dee, and E. F. Knol. 2022. Novel experimental design reveals additional evidence of natural genetic variation in response to PRRS challenge. Proc. NAPRRS/NCR229 International Conference of Swine Viral Diseases, page 50. Chicago, Illinois. December 2-4, 2022.

[CIT0012] Falconer, D. S. 1954. Asymmetrical responses in selection experiments. In Symp. Genet. Popul. Struc. Int. Union Biol. Sci. Naples, Series B. 15:16–41. doi:10.1038/hdy.1968.69

[CIT0013] Faure, J., L. Lefaucheur, N. Bonhomme, P. Ecolan, K. Meteau, S. M. Coustard, M. Kouba, H. Gilbert, and B. Lebret. 2013. Consequences of divergent selection for residual feed intake in pigs on muscle energy metabolism and meat quality. Meat Sci. 93:37–45. doi:10.1016/j.meatsci.2012.07.006 22910803

[CIT0014] Gilbert, H., Y. Billon, L. Brossard, J. Faure, P. Gatellier, F. Gondret, E. Labussière, B. Lebret, L. Lefaucheur, N. Le Floch, et al. 2017. Review: divergent selection for residual feed intake in the growing pig. Animal. 11:1427–1439. doi:10.1017/S175173111600286X 28118862 PMC5561440

[CIT0015] Gómez-Laguna, J., F. J. Salguero, F. J. Pallarés, and L. Carrasco. 2013. Immunopathogenesis of porcine reproductive and respiratory syndrome in the respiratory tract of pigs. Vet. J. 195:148–155. doi:10.1016/j.tvjl.2012.11.012 23265866 PMC7128372

[CIT0017] Hermesch, S., L. Li, A. B. Doeschl-Wilson, and H. Gilbert. 2015. Selection for productivity and robustness traits in pigs. Anim. Prod. Sci. 55:1437–1447. doi:10.1071/an15275

[CIT0019] Holtkamp, D. J., J. B. Kliebenstein, J. J. Zimmerman, E. Neumann, H. Rotto, T. K. Yoder, C. Wang, P. Yeske, C. L. Mowrer, and C. Haley. 2013. Assessment of the economic impact of porcine reproductive and respiratory syndrome virus on US pork producers. J. Swine Health Prod. 21:72–84. doi:10.2460/javma.2005.227.385

[CIT0020] Hume, D. A., C. B. A. Whitelaw, and A. L. Archibald. 2011. The future of animal production: improving productivity and sustainability. J. Agric. Sci. 149:9–16. doi:10.1017/s0021859610001188

[CIT0021] Lager, K. M., W. L. Mengeling, and S. L. Brockmeier. 1999. Evaluation of protective immunity in gilts inoculated with the NADC-8 isolate of porcine reproductive and respiratory syndrome virus (PRRSV) and challenge-exposed with an antigenically distinct PRRSV isolate. Am. J. Vet. Res. 60:1022–1027. doi:10.2460/ajvr.1999.60.08.1022 10451216

[CIT0022] Li, Q., and J. F. Patience. 2017. Factors involved in the regulation of feed and energy intake of pigs. Anim. Feed Sci. Technol. 233:22–33. doi:10.1016/j.anifeedsci.2016.01.001

[CIT0025] Márquez, G. C., P. B. Siegel, and R. M. Lewis. 2010. Genetic diversity and population structure in lines of chickens divergently selected for high and low 8-week body weight. Poult. Sci. 89:2580–2588. doi:10.3382/ps.2010-01034 21076095

[CIT0026] Meng, X. J. 2000. Heterogeneity of porcine reproductive and respiratory syndrome virus: implications for current vaccine efficacy and future vaccine development. Vet. Microbiol. 74:309–329. doi:10.1016/s0378-1135(00)00196-6 10831854 PMC7117501

[CIT0027] Merks, J. W., P. K. Mathur, and E. F. Knol. 2012. New phenotypes for new breeding goals in pigs. Animal. 6:535–543. doi:10.1017/S1751731111002266 22436267

[CIT0028] Mpetile, Z. 2014. Effect of divergent selection for residual feed intake on immune system of Yorkshire pigs [PhD dissertation]. Iowa State University, Ames.

[CIT0029] Mulder, H. A., and H. Rashidi. 2017. Selection on resilience improves disease resistance and tolerance to infections. J. Anim. Sci. 95:3346–3358. doi:10.2527/jas.2017.1479 28805915

[CIT0030] Neumann E. J. , J. B. Kliebenstein, C. D. Johnson, J. W. Mabry, E. J. Bush, A. H. Seitzinger, A. L. Green, J. J. Zimmerman. 2005. Assessment of the economic impact of porcine reproductive and respiratory syndrome on swine production in the United States. J. Am. Vet. Med. Assoc. 227:385–392. doi:10.2460/javma.2005.227.385 16121604

[CIT0032] Nielsen, M. K., L. D. Jones, B. A. Freking, and J. A. DeShazer. 1997. Divergent selection for heat loss in mice: I. Selection applied and direct response through fifteen generations. J. Anim. Sci. 75:1461–1468. doi:10.2527/1997.7561461x 9250505

[CIT0033] Niemi, J. K., M. L. Sevón-Aimonen, A. H. Stygar, and K. Partanen. 2015. The economic and environmental value of genetic improvements in fattening pigs: an integrated dynamic model approach. J. Anim. Sci. 93:4161–4171. doi:10.2527/jas.2015-9011 26440196

[CIT0034] Pineiro, C., J. Morales, A. Dereu, N. Wuyts, O. Azlor, E. Vizcaino, and P. Doncecchi. 2014. Individual Pig Care program improves productive performance and animal health in nursery-growing pigs. J. Swine Health Prod. 22:296–299. doi:10.54846/jshap/831

[CIT0035] Rauw, W. M. 2012. Immune response from a resource allocation perspective. Front. Genet. 3:267. doi:10.3389/fgene.2012.00267 23413205 PMC3571735

[CIT0036] R Core Team. 2021. R: a language and environment for statistical computing. R Foundation for Statistical Computing, Vienna, Austria. [accessed August 10, 2022]. https://www.R-project.org/

[CIT0037] Rowland, R., J. Lunney, and J. Dekkers. 2012. Control of porcine reproductive and respiratory syndrome (PRRS) through genetic improvements in disease resistance and tolerance. Front. Genet. 3:260. doi: 10.3389/fgene.2012.00260 23403935 PMC3565991

[CIT0039] Yu, J., J. Wu, Y. Zhang, L. Guo, X. Cong, Y. Du, J. Li, W. Sun, J. Shi, J. Peng, et al. 2012. Concurrent highly pathogenic porcine reproductive and respiratory syndrome virus infection accelerates *Haemophilus parasuis* infection in conventional pigs. Vet. Microbiol. 158:316–321. doi:10.1016/j.vetmic.2012.03.001 22460022

